# Antibiotic use and antimicrobial resistance: Knowledge, Attitude and Practices survey of medical students to evaluate undergraduate training curriculum

**DOI:** 10.1099/acmi.0.000638.v4

**Published:** 2025-01-08

**Authors:** Rushika Saksena, Annapurna Parida, Madhura Jain, Rajni Gaind

**Affiliations:** 1 Vardhman Mahavir Medical College and Safdarjung Hospital, New Delhi, India

**Keywords:** antimicrobial resistance, KAP survey, undergraduate medical students

## Abstract

**Introduction.** A better understanding of knowledge, attitude and practices of undergraduate medical students towards antimicrobial resistance (AMR) is necessary to identify gaps in the current training curriculum.

**Methods.** A 20-point Likert scale-based questionnaire divided into three parts, knowledge, attitude and practices, relating to antibiotic use and resistance was devised. Students attending each year of the undergraduate medical programme were approached to participate in the study over a 1-week period. Knowledge, Attitude and Practices scores of each year were compared through logistic ordinal regression and the Kruskal–Wallis (KW) test.

**Results.** Two hundred and eight students participated in the study. Overall, knowledge of about intended use of antibiotics, fixed drug combinations and awareness about AMR was good (average score of 73.75%). Steady improvement in knowledge scores was observed from the first year (−0.441) to the final year (0.00). The medical students had favourable attitude towards rational antimicrobial use (Likert score ≥4), including the need to spread awareness about AMR amongst students and the public and following doctor’s prescriptions. Self-medication was reported by 28.4% of students and hoarding of leftover doses by 49.1%. Attitude score had a direct correlation with the knowledge score on the KW test (*χ*^2^=29.6, *P*≤0.5) but had no significant correlation with antimicrobial practices (*χ*^2^=3.9, *P*≥0.5). The gaps identified in students’ practices included self-medication, skipping of dosing and hoarding of leftover medication.

**Conclusion.** As improvement in knowledge did not correlate with better personal behaviours regarding antibiotics, the current curriculum needs to include AMR as a focus area to ensure good antibiotic prescribing practices in future practitioners.

## Data summary

The authors confirm that all supporting data have been provided within the article as figures and tables or through Excel file with the article.

## Introduction

Antimicrobial resistance (AMR) is a silent pandemic, which adversely impacts patient care with an increase in both direct and indirect economic costs, morbidity and mortality in both hospital and simple community-acquired infections [1]. The World Health Organization (WHO) has recognized rationalizing antibiotic use through modification in prescription practices and control of over-the-counter purchases as a few measures to curtail antibiotic use. This needs a behavioural change on the part of the practitioner as well as the community [2]. It also recommended training and education of healthcare workers including medical students on rational antimicrobial use as an integral part of AMR containment strategy [3]. India is one of the largest consumers of antibiotics globally with an increase of 103% from 2000 to 2015 including those from the WHO Watch group of antibiotics [4,5]. India also accounts for a large burden of AMR with an alarmingly high level of resistance to reserve antibiotics like carbapenems [6].

Unlike developed countries, India does not have ‘infectious diseases’ as a recognized specialty with only a few medical colleges offering a structured course in infectious diseases as a postgraduate programme [7]. Medical students after undergraduate training are expected to diagnose and manage patients, which includes prescribing antibiotics under minimal specialized supervision. A number of studies across the world have shown poor knowledge and practices regarding antibiotic use and prescription amongst medical students [8,11]. Some countries like the UK have published consensus-based competencies for teaching antimicrobial resistance and stewardship to undergraduate medical students [12]. Various novel approaches have been used to teach the appropriate use of antimicrobials at the undergraduate medical level with varying success [13,14].

The present study was designed to assess the knowledge, attitude and practices regarding antimicrobial resistance and good antibiotic practices of undergraduate medical students in a large tertiary care teaching hospital. The study was aimed at identifying the present gaps that can be addressed in the revised medical curriculum.

## Methods

### Setting

Our hospital was established in 1942 and affiliated to the medical college in 2002. Every year, 180 students are admitted to the undergraduate medical (MBBS) course. The students are selected from across India through a national-level entrance test. MBBS (Bachelor of Medicine and Bachelor of Surgery) in India encompasses 4.5 years of study and 1-year internship. Information such as gender and socioeconomic background were not collected as part of this survey. The undergraduate course includes the study of basic sciences in the first year, training in allied sciences in the second and third years and clinical rounds with bedside teaching commencing from the second year. A cross-sectional study was conducted in August 2019 over a 1-week period to understand the perception of students towards antimicrobial resistance. The study was approved by the institutional ethics committee (No. IEC/SJH/VMMC/Project/August-2017/990).

### Questionnaire

A pre-designed, 20-point questionnaire divided into three parts, knowledge, attitude and practices, was used for the collection of data. Part I focused on knowledge regarding antibiotics and antibiotic resistance and consisted of seven dichotomous questions with yes or no answers and one multiple-choice question. Part II and Part III consisted of six questions based on the 5-point Likert scale. Part II was designed to assess attitudes towards the judicious use of antibiotics with responses varying from strongly disagree to strongly agree. Part III assessed the antibiotic practices of students with answers ranging from never to always. The questionnaire was vetted by experts before starting the study. Cronbach’s alpha (an estimate of internal consistency and scale reliability) was 0.90. Scores were created by summing the scores for respective items such that a higher score indicated more positive knowledge.

The questionnaires were distributed in classrooms after a lecture. All ongoing MBBS batches were surveyed within a time frame of 1 week. Interns were not included as they can be considered practitioners with licenses to prescribe medication in a limited capacity within the hospital. Medical students from first year to final year belonged to an age range of 18–24 years. Participation in the study was purely voluntary and anonymous. A total of 211 questionnaires were collected; however, three of these were incomplete and were excluded from the analysis. Of the 208 MBBS students included from the first year to final year, ~42 students were enrolled from each year of the course. The overall response rate was 23.4%.

### Statistical analysis

Data were coded and analysed using SPSS 16 (RRID:SCR_002865). Descriptive statistics were used to summarize the numerical variables (mean) and categorical variables (expressed as frequencies and percentages). Ordinal regression was applied for categorical variables to test for significance. Continuous variables were assessed for statistical significance using the Kruskal–Wallis one-way ANOVA on Ranks.

## Results

### Knowledge

Table 1 shows the results of the knowledge survey of the medical students regarding antibiotics, their use and resistance during the different phases of the MBBS curriculum. The majority of our students surveyed during the study period were aware of antibiotic resistance (92–100%). Knowledge of other aspects of antibiotics such as their efficacy in bacterial rather than viral infections, the effect of antibiotic use on normal bacterial flora and awareness about AMR as a concept was also correctly understood by the majority of students (92.4–99.5%) (Table 1). Only 7.6% (*n*=16) of our medical students believed that antibiotics could be used to treat viral infections, and even fewer (2.4%, *n*=5) responded that antibiotics should be taken for all febrile episodes. The majority of students (95.7%, *n*=199) were aware that antibiotics create an imbalance in the normal microbial flora.

**Table 1.** Knowledge of medical students about antibiotic use and antimicrobial resistance (percentage of respondents answering ‘yes’)

Question	First year (*n*=40) (%)	Second year (*n*=47) (%)	Third year (*n*=40) (%)	Fourth year (*n*=40) (%)	Fifth year (*n*=41) (%)	Total (*n*=208) (%)
Have you ever heard of bacterial resistance to antibiotics?	92.5	97.9	100	100	97.5	98.1
Do you think antibiotics can be used to treat bacterial infections?	97.5	100	100	100	100	99.5
Do you think antibiotics can be used to treat viral infections?	0	6.4	12.5	5	14.6	7.7
Do you think antibiotics should be taken every time you have fever?	7.5	4.3	0	0	0	2.4
Do you think antibiotics can cause an imbalance in the body’s normal bacterial flora?	90	89.36	100	100	97.5	95.7
Have you ever heard of fixed-drug combinations?	32.5	55.3	95	95	92.7	73.6
Do you think humans can become resistant to antibiotics?	65	59.6	20	20	36.6	40.9

It was heartening to find that awareness about the fixed-dose antibiotic combination was high (73.5%, *n*=153) amongst our students and in fact increased year-on-year from 32.5 to 92.7%. A large percentage of students (40.9%) believed that humans become resistant to antibiotics rather than bacteria; however, this misconception decreased over the years (Table 1) from 65 to 36.5% (*P*-value=0.01). Further, a majority of students (84%) identified abuse in humans as the most important cause of the spread of antibiotic resistance.

Knowledge of medical students on all aspects of antimicrobial resistance was good overall, with an average score of 73.75% (5.9/8). On the ordinal regression scale, the overall knowledge score showed steady improvement from the first year (−0.441) to the final year (0.00) medical students with *P*-value ≤0.5.

### Attitude

**Fig. 1.**
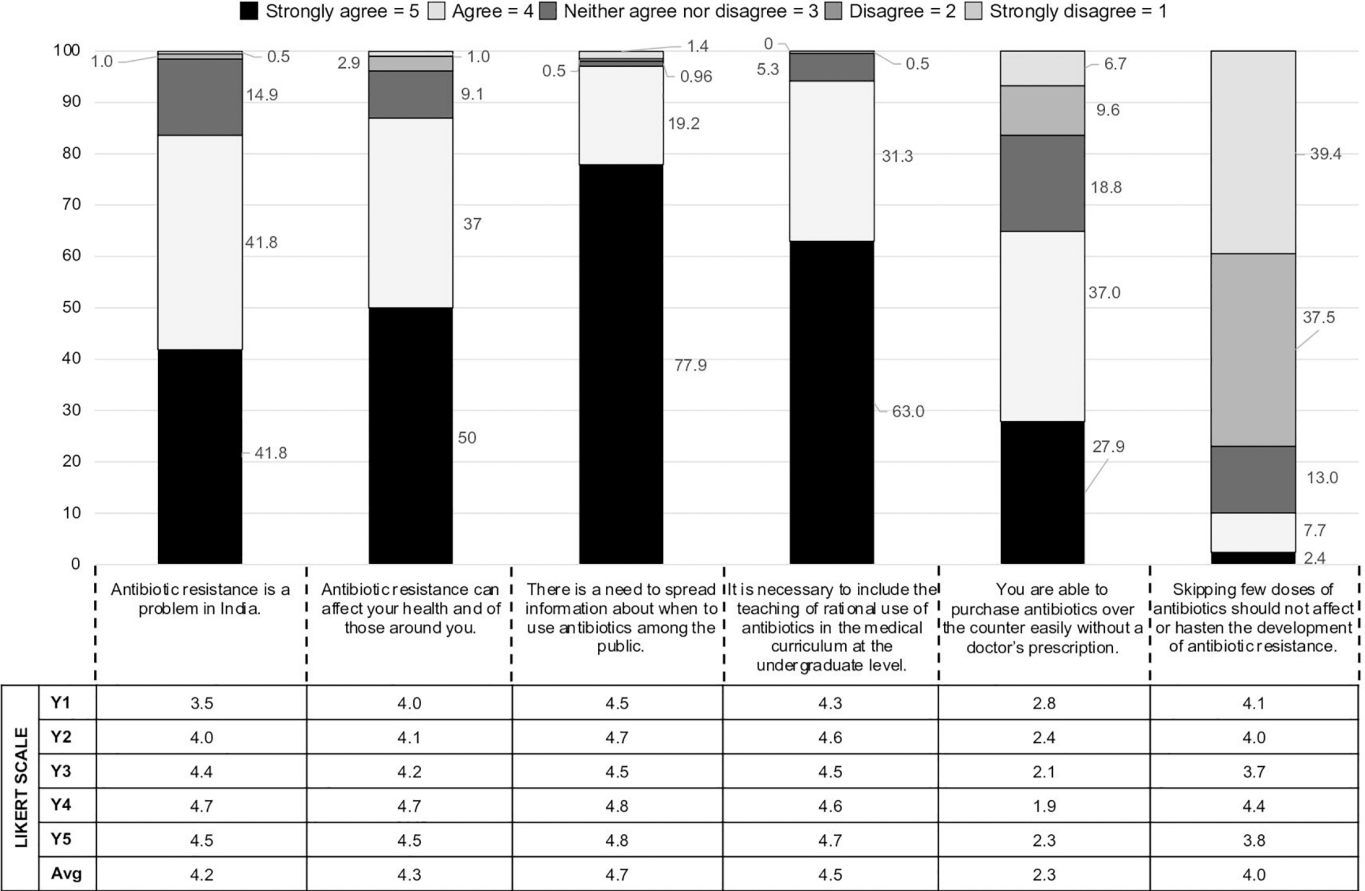
Year-on-year comparison of the attitude of medical students towards antibiotic use and antimicrobial resistance (in percentage and Likert scores).

In the present study, the attitude of students towards antimicrobial use was favourable with score of 4 or more on the Likert scale (LS) across the years of study (Fig. 1). Amongst our students, 85% (*n*=177) recognized antibiotic resistance as a problem that could adversely affect their health (87%, *n*=181). An overwhelming majority of students favoured the need to spread public awareness about AMR (97%, *n*=202) as well as increased focus on AMR in undergraduate teaching (94%, *n*=195). We found that 65% of students acknowledged that they are able to purchase antibiotics without a prescription and 77% agreed that skipped doses hasten the development of antibiotic resistance.

### Practices

**Fig. 2.**
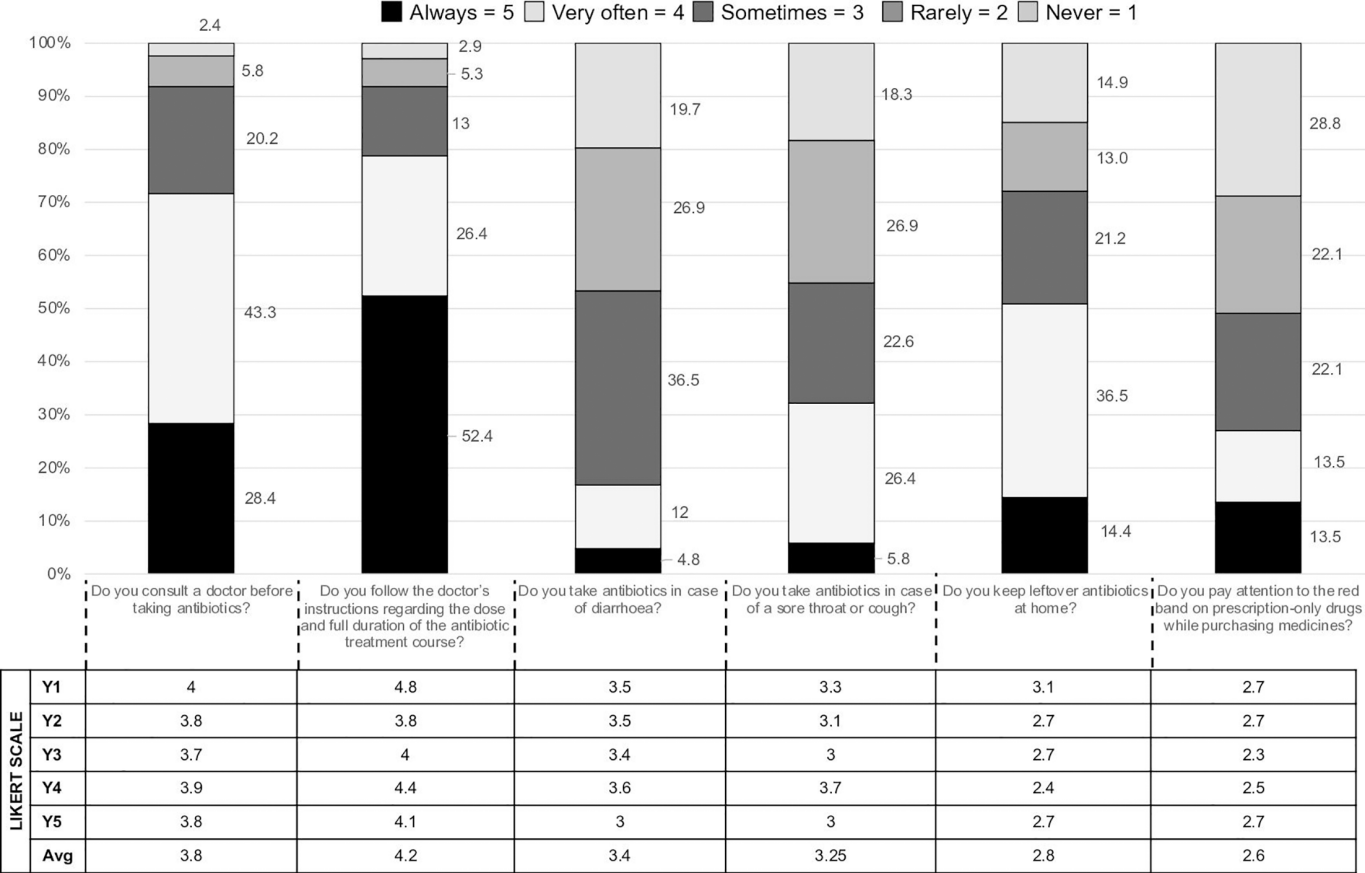
Year-on-year comparison of practices of medical students regarding antibiotic use (in percentage and Likert scores).

Likert scores for questions regarding antimicrobial practices fell behind those for attitude on an average by 2.1–3.4 points; however, an average Likert score remained consistent over the years (Fig. 2). Overall response to the question on self-medication was satisfactory with an average Likert score of 3.8. Only 28% of students said they always consult a doctor before taking antibiotics, whereas 43% said that they did very often but not always (Fig. 2). Self-medication was more evident in older students (average LS ≤3 for both sore throat and diarrhoeal diseases) than first-year students (first year, LS ≥3.5 for sore throat and 3.3 for diarrhoea). Overall, students tend to take antibiotics for sore throat more commonly than for diarrhoea (Fig. 2).

Over half of the surveyed students (50.9%) affirmed skipping doses as well as hoarding antibiotics at home (LS 2.4–3.1). Only 13.5% students acknowledged that they always pay attention to the red line on packaging. This practice was observed uniformly over the years with LS=2.7 in first year as well as in final-year students (average LS=2.6).

To know the correlation between knowledge and attitude as well as the knowledge and practices of students, the Kruskal–Wallis one-way ANOVA on Ranks test was performed. The total attitude and total practice scores of students were calculated by summation of individual Likert scores for each response. Favourable responses were awarded a higher score. The maximum attitude score and practice score that could be achieved were 30 each. Knowledge scores had no correlation to attitude score (*P*-value=0.082) or practice score (*P*=0.698).

## Discussion

### Knowledge

In our study, >92% of our students were aware of antibiotic resistance. They understood that antibiotics were effective only in treating bacterial rather than viral infection and their effect on normal bacterial flora. Our findings are in concordance with the knowledge of antibiotics and antimicrobial resistance amongst medical students from Italy (95.2%) and China (92.9%) [11,15]. In contrast, Suaifan *et al*. reported comparatively low (70.4%) awareness of AMR amongst medical students from Jordan [16].

In comparison to these three studies, where a higher percentage of students (17–35%) were reportedly unaware of the inefficiency of antibiotics for viral illnesses, only 7.6% (*n*=16) of our medical students believed that antibiotics could be used to treat viral infections [11,15, 16]. In countries like India, many febrile illnesses are caused by viruses like dengue, Japanese encephalitis, seasonal flu and chikungunya, which are self-limiting and do not need antibiotic therapy. In this context, it was interesting that student awareness about the inefficiency of antibiotics for viral illnesses was higher than in other countries. A mere 2.4% of our students responded that antibiotics could be used to treat every febrile episode, which is similar to the Italian (1.8%) study [11]. In contrast, the Chinese and Jordanian studies reported that 22–50% of medical students agreed that antibiotics could be used for every febrile episode [15,16]. The WHO has reported a common misconception amongst general public (76%) that humans develop resistance to antibiotics rather than bacteria [17]. Nearly 40% (*n*=85) of students in our study also held this belief in comparison to the Italian study where only 7% were unclear on this concept [11]. Antibiotic use creates an imbalance in the microbial flora and thus selects resistance [18,20]. Medical students in our study were largely (95.7%, *n*=199) aware of this fact like the Italian study (90.2%) [11]. Although all the above studies were conducted in undergraduate medical students, knowledge and awareness regarding antibiotic use and resistance vary geographically likely due to differences in the curriculum of medical education. The differences may also be attributed to the framing of questions.

Fixed-dose combination (FDC) consists of two or more approved drugs combined in a single dosage form in a fixed ratio, manufactured and distributed in specified doses to treat either a single ailment or multiple comorbid conditions [21]. Their commercial success has led to introduction of many irrational newer combinations (e.g. norfloxacin+metronidazole, amoxicillin+dicloxacillin, cefixime+linezolid and cefuroxime+linezolid). These irrational FDCs often have pharmacokinetic and pharmacodynamic mismatch associated with reduced efficacy and enhanced toxicity [22]. The fixed-dose antibiotics may not contain effective doses of individual drugs leading to mutations and selection pressure proliferating resistant strains [20]. On 14 September 2018, the government of India banned 328 irrational FDCs on the recommendation of the Drug Technical Advisory Board [23]. It was heartening to find that awareness about the fixed-dose antibiotic combination was high (73.5%, *n*=153) amongst our students.

**Fig. 3.**
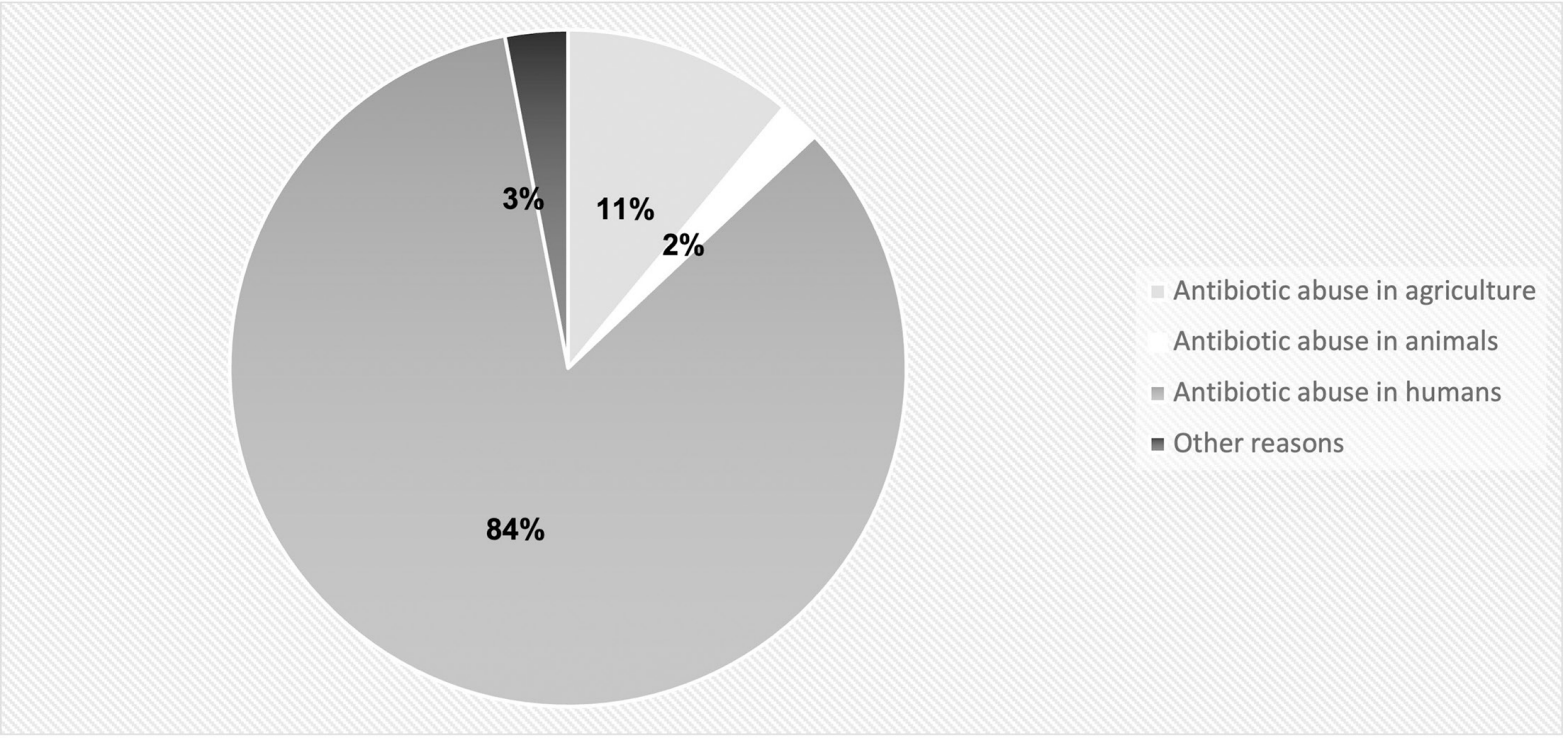
Response of students to the question ‘What do you think is the most common cause of the spread of antibacterial resistance?’.

Widespread antibiotic use in livestock and agriculture has led to rapid emergence and dissemination of AMR [2,24]. Most of this use involves the addition of low-dose antibiotics to animal feed as growth promoters or at higher doses for disease prevention. We found that most students did not think that abuse of antibiotics in animals and agriculture was a major contributor to AMR, with an overwhelming 84% identifying abuse in humans as the most important cause (Fig. 3)

Although the knowledge about antibiotic resistance and use was similar amongst students in different years of the course, the improvement was observed in two areas, i.e. awareness about fixed-dose combinations and misconception about humans becoming resistant to antibiotics. The third and fourth years of medical education in India is more clinically oriented with a focus on patient diagnosis and management. Moreover, they are trained in prescribing antibiotics for syndromic management of infections, surgical prophylaxis etc. This clinical exposure of students has a positive impact and helps clarify many preformed notions and prejudices amongst students regarding antibiotics over time [11,25].

### Attitude

The survey showed that a consistently favourable score of ≥4 over the school years indicates that most students understood the importance of antimicrobial resistance and rational antimicrobial use. In a multicentre European study on 338 medical students, 92% had agreed that AMR is a national problem in their country [25]. Similarly, we found that 83.6% of our students agreed with this statement. Previous studies from Malaysia (88%), the USA (75%) and China (89%) had reported that most medical students felt the need for more comprehensive teaching on antibiotics for better prescribing practices [15,26, 27]. We found similar responses from our students, perhaps highlighting that the challenge of AMR and its awareness amongst the public and medical fraternity is a concern recognized by medical students across the globe.

With reports of unregulated pharmacies and unethical practices, and the availability of over-the-counter antibiotics, antibiotic misuse in India is now staggering. This was reflected in our study, where two-thirds (65%) of medical students (Likert score 2.3) agreed that antibiotics can be bought without a valid prescription. Seventy-seven per cent of our students identified that skipping doses contributes to development of AMR. This is higher than a previous Indian study (57% of students) published in 2013 [28]. AMR awareness has been prioritized in the undergraduate curriculum under the national action plan on AMR containment notified by the government of India in 2017. This has likely resulted in better scores in comparison to previous reports from India. However, in the Jordanian study, 84.3% of medical students were able to identify skipping of doses as a contributing factor for AMR [16].

### Practices

Good antibiotic practices include taking antibiotics after a doctor’s consultation, following the dosing schedule and avoiding self-medication [29]. James et al had reported medical students were prone to self-medication as well as irrational drug usage [29]. Upper respiratory tract ailments and diarrhoea were the most common illnesses for which people tend to self-medicate. However, the most common causative organisms in both these ailments are viruses, and antibiotics hardly have any role in treatment.

In contrast, in a previous report from India by Afzal *et al.* in 2013 where only 10% of medical students admitted to self-medicate with antibiotics, in our study, 28.4% admitted to self-medication at least sometimes [28]. Similar findings were seen in the Chinese study where the common cold was the leading cause of self-medication; a third of our students (33.2%) tended to self-medicate often in case of an upper respiratory tract infection [15].

Skipping doses and buying more than required quantity add to another problem of ‘leftover antibiotics’. The WHO had recommended that leftover antibiotics should neither be used nor shared without consulting doctors first. Afzal *et al*. have reported that 41% students had admitted having skipped doses, 36% agreed they never discard left-over antibiotics, and above 50% had shared these antibiotics with family and friends [28]. Similarly, half of our students also admitted that they skip doses and horde the leftover medicines. Although there is a year-on-year increase in tendency for self-medication amongst students, it is also accompanied by better adherence to the dosing schedule. Previous studies have attributed clinical training of older students in disease aetiology and management which enables them to diagnose and self-treat many illnesses [15,16].

On 1 March 2014, Schedule H1 notification came into force with the intent to control the rampant misuse and over-the-counter sale of antibiotics [30]. Schedule H1 stipulates the retail dispensing of drugs only against a valid prescription. Currently, 46 drugs have been placed under this restricted category, which mainly comprises third- and fourth-generation cephalosporins, carbapenems, newer fluoroquinolones and first- and second-line antitubercular drugs. The packaging of these drugs has a mandatory Schedule H1 warning printed on the label in a box with a red border and the Rx symbol in red [30]. Just 13.5% of students paid attention to the red line on packaging while buying medication. This is a unique finding of our report as previous studies have not explored this aspect of antibiotic use.

In a study conducted on high school students and teachers in Delhi, it was found that students had poor knowledge about antibiotic use and resistance, while the teachers had only a basic understanding [31]. Our first year students represent this population, and we can see a gradual increase in knowledge scores of medical students. However, knowledge scores did not match the attitude and practice scores. Previous studies have also highlighted that students do not practice what they know [11,15, 16, 28]. This gap highlights that the current curriculum improves the knowledge of students; however, it is not enough to change the attitudes and practices. To improve antibiotic practices, innovative teaching-learning methods which focus on psychomotor (clinical skills) and affective domain (behavioural change) including AETCOM (attitude, ethics and communication) modules need to be introduced at this level of medical education. These may include regular workshops in prescribing practices, role plays, competitions etc. Students should be encouraged to actively participate, and feedback from the students should be obtained for further improvement to the curriculum.

## Conclusion

Knowledge of medical students regarding AMR steadily improved over the years of study. However, some incorrect concepts and practices, like misconceptions about the development of AMR, self-medication, skipping of dosing and hoarding of leftover medication, formed in the first year persist through their final year. The present study highlights a lack of correlation between knowledge, attitude and practices amongst Indian medical students. As improvement in behaviour lagged behind that in knowledge, the authors conclude that the current curriculum is unable to change the practices of students. With the introduction of revised competency-based medical education (CBME) for undergraduate medical students in India, it is hoped that the above gaps will be bridged.

Poor response rates are commonly reported amongst students in surveys of medical education. This was also observed in our study and may have led to responder bias. A follow-up study after the introduction of CBME for Indian medical graduates is needed to see the changes in Knowledge, Attitude and Practices scores of the students over time.

## supplementary material

10.1099/acmi.0.000638.v4 Uncited Table S1.

## Abbreviations

AMR: antimicrobial resistance
CBME: revised competency-based medical education
FDC: fixed-dose combination
KAP: Knowledge Attitude Practices
KW: Kruskal–Wallis
LS: Likert scale
MBBS: Bachelor of Medicine and Bachelor of Surgery
WHO: World Health Organization
